# Pacing for Patients Suffering From Cardioinhibitory Vasovagal Syncope Using the Closed-Loop System

**DOI:** 10.3389/fcvm.2019.00192

**Published:** 2020-02-14

**Authors:** Gonzalo Barón-Esquivias, Carmen Barón-Solís, Antonio Ordóñez

**Affiliations:** ^1^Cardiology and Cardiac Surgery Department, Virgen del Rocio University Hospital, Seville University, Seville, Spain; ^2^Instituto de Biotecnología de Sevilla (IBIS), Seville, Spain; ^3^Centro de Investigacion en Biomedicina en Red Cardiovascular (CIBER-CV), Madrid, Spain; ^4^Centro Superior de Investigaciones Científicas (CSIC), Madrid, Spain

**Keywords:** vasovagal syncope, cardioinhibition, pacing, rate-drop-response, closed-loop system, syncope

## Abstract

One in three vasovagal syncope (VVS) patients has syncopal recurrence after diagnosis, despite the standard recommendations for the avoidance of a recurrence, and one in five patients has more than one syncopal recurrence in the medium term. Given the high prevalence of VVS, there is a large population that continues to need effective treatment. There are numerous studies that use the implantable loop recorder (ILR) to document a cardioinhibitory response during VVS, with one study, ISSUE-3, demonstrating the efficacy of pacing using the rate-drop-response algorithm to trigger pacing and prevent syncopal recurrence in this population. There are more uncertainties in the studies that have used head-up tilt test (HUT) to select the population for pacing. We have recently performed the SPAIN randomized, controlled clinical trial using HUT to select the patients for pacing. The conclusion of the study was that, with the closed-loop system to introduce pacing, there was a significant reduction in the burden of syncope and a seven-fold increase in the time to first recurrence of syncope, which was greater than in the ISSUE-3 study. Since the completion of the SPAIN trial and its inclusion in the European guidelines, in our daily clinical practice, the use of this therapy is still recommended with caution in the context of the available literature, but it has increased our confidence in so doing. One in five patients with VVS needs treatment because of a high syncopal load. If an ILR is used to select the patients for pacing, the rate-drop-response algorithm can be recommended. In patients who have asystole on HUT, pacing with the closed-loop system has higher success and must now be considered as a tenable option for VVS patients.

## Introduction

Vasovagal syncope (VVS) is generally considered as a benign disease. Up to 40% of the population experience at least one syncope in a lifetime, with most patients having no more than a single episode (1). Considering the patients who are referred to cardiologists, their number of syncopes is typically three, ranging from one to five episodes, and in those with recurrent episodes, their quality of life is reduced (2, 3). The most important aspect of the management of these patients is to explain and reassure them about what happens in an episode and to emphasize physical counter-measures and changes in lifestyle (4). Although no clinical studies have compared these recommendations with controls, there is a consensus that they have a beneficial effect in reducing the syncopal recurrences (4). After diagnosis, a third of patients presenting to specialized syncope facilities have recurrences, and one in five patients have more than one recurrence, which implies that 14% require some additional treatment beyond the standard measures (4, 5). The most pressing cases requiring additional treatment are those who have recurrent syncope with short or absent prodromes and those who sustain VVS during high-risk activities.

The studies of pacing in VVS using head-up tilt test (HUT) for patient selection were published in the 1990s and early 2000s, while findings for those guided by implantable loop recorder (ILR) followed. Both sets of studies served to deepen the knowledge of VVS. Cardioinhibition observed during induced and spontaneous VVS prompted the use of pacemakers (PMs) as treatment for these patients. However, the use of HUT to decide on the necessity of pacing is actually in doubt based on published data (6, 7). The number of VVS patients treated by pacing based on HUT findings has fallen substantially. It was frequent in the 1990s, but it has reduced to be exceptional today. A study by our group conducted between 1990 and 2000 reported PM implantation in 58 (17.5%) of 330 patients with recurrent VVS and positive HUT (8). Additionally, in a Swedish study conducted between 2008 and 2016, only 41 (4.4%) of 933 patients with VVS and positive HUT received a pacemaker as the preventive syncope treatment (9).

## VVS Treatment Based on ILR Results

The introduction of ILR as a diagnostic tool opened the doors to the design of studies that, based on ILR findings, selected pacing as a therapy for these patients. The International Study of Syncope of Unknown Etiology (ISSUE) series of studies included patients with syncope and documentation of spontaneous cardioinhibition on ILR during syncope; they were thus selected for pacing. In ISSUE-2, the recurrence per year in 53 patients who received pacing therapy was 10% compared with 41% in patients without specific therapy (80% reduction in relative risk for patients, *p* = 0.002, and 92% for syncope burden, *p* = 0.002) (10). The 1-year recurrence rate in patients with pacemakers was 5%. This study was a registry rather than a randomized, double-blind, controlled trial (10). ISSUE-2 thus prompted ISSUE-3 (11), which was designed as a multicenter, prospective, randomized, and double-blind trial to evaluate the effectiveness of dual-chamber pacing (DDD) with the rate-drop-response algorithm (RDR) to prevent the recurrence of syncope. These patients, aged >40 years, presented documented asystole in the spontaneous ILR recordings of VVS. Seventy-seven patients were randomly assigned to DDD–RDR stimulation or to the group who will have only sensing without pacing. The recurrence of syncope during follow-up occurred in 27 patients, 19 of whom had been assigned to the sensing mode and eight to the active pacing. At 2 years, syncope recurrence occurred in 57% with an implanted device in sensing mode and in 25% with active pacing, representing 57% reduction in recurrence. ISSUE-3 was the first trial with a strong design to show the pacing benefit in VVS. These findings were used to justify the Class IIa indication for pacing in patients >40 years old who suffer from recurrent VVS and have documented asystole on ILR during spontaneous VVS (4, 12).

However, the use of pacing bases its effectiveness on the fact that the patient suffering from VVS has a predominant cardioinhibition since it is not anticipated to be effective in preventing vasodilation and hypotension. In a substudy of ISSUE-3, an asystolic response during HUT predicted asystole during spontaneous syncope as documented by ILR, with a positive predictive value of 86% (13). A meta-analysis including four studies on patients with syncope and documented asystole on ILR showed that the benefit of pacing was less in those patients who had a positive response during HUT although the confidence interval was large (13–53%), preventing a definitive conclusion regarding the benefit of pacing in these patients (14).

**Figure 1:**
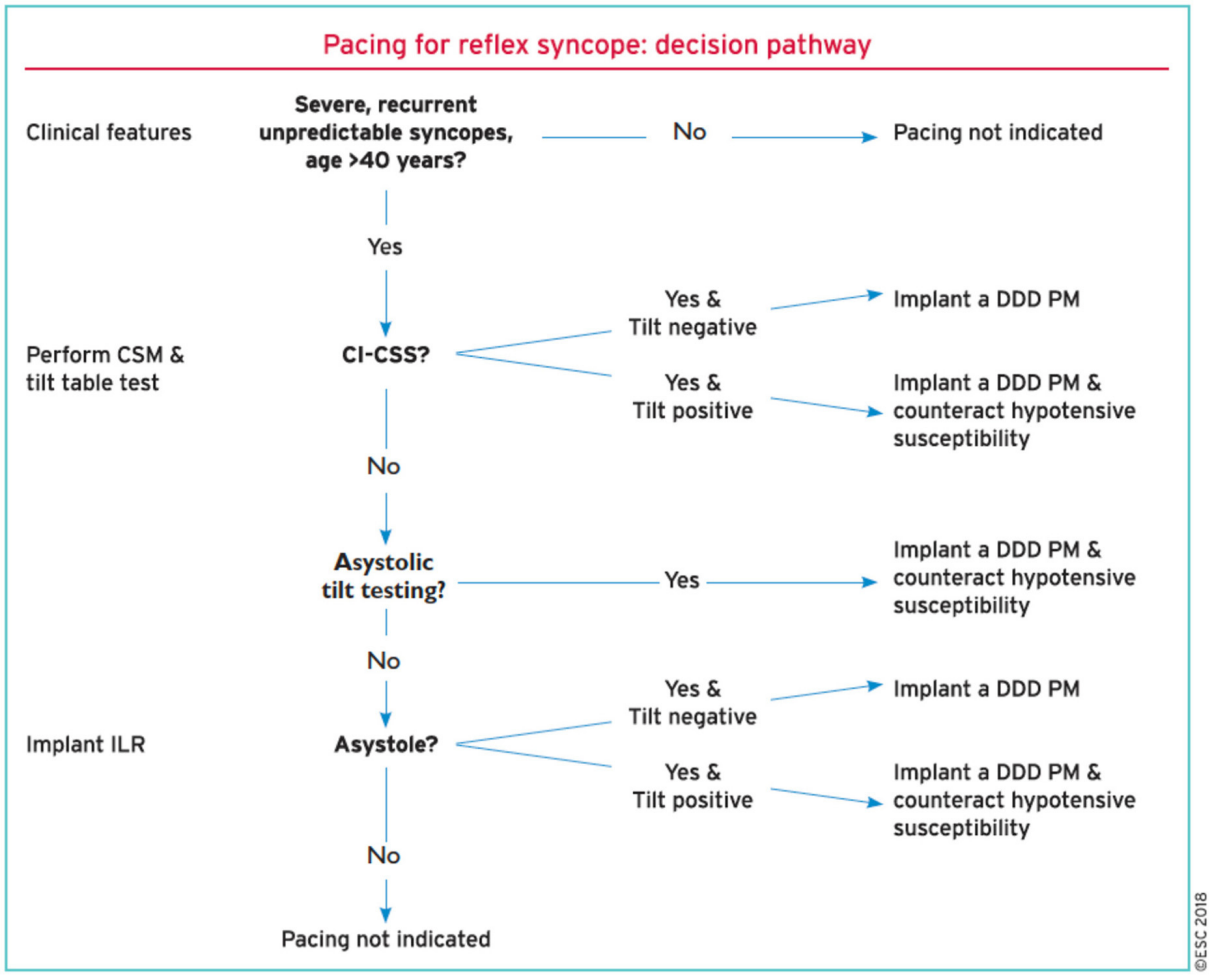
An algorithm decision tree for the selection of patients with severe recurrent vasovagal syncope who are eligible for pacemaker therapy as recommended by the European guidelines (4).

Finally, in the SUP-2 study (5, 15), an Italian registry study from 10 syncope units employing a uniform algorithm for the management of older patients (mean age 73 years) with clinically likely reflex syncope, in those patients undergoing HUT, 38 of whom had a dominant cardioinhibition (mean asystole of 22 ± 16 s), the syncopal recurrence after pacing was 3% at 1 year, 17% at 2 years, and 23% at 3 years. These percentages were less than those observed in the untreated patients in the study. The strategy of the SUP-2 study consisted of three progressive steps based on recent guidelines: first, the carotid sinus massage in which, if positive with cardioinhibition, pacing was selected; second, HUT, where if positive likewise with cardioinhibition, pacing was selected; third, ILR, where again if positive with cardioinhibition, pacing was chosen, and if not positive or if cardioinhibition is absent, ILR monitoring was continued (4, 15, 16) (Figure 1).

## Vasovagal Syncope Treatment With Closed-Loop Stimulation

It is well-known that the physiological sensors in pacemakers can optimize their function (17–20). The so-called closed-loop system (CLS) sensor tracks the variations in intracardiac (right ventricular) impedance during the systolic phase of the cardiac cycle (21). These changes in intracardiac impedance are closely correlated both with the right and left ventricular d*P*/d*t* and right ventricular volume, making this system a detector of both the contractility and the right ventricular volume in the early phase of VVS (22, 23). The first study looking at “neuromediated inotropic pathophysiology” showed a significant increase in heart contractility in nine patients in the minutes preceding the HUT-induced neurally mediated syncope (NMS), also corroborated by what is known concerning the epinephrine rise in this period (24–26). It was suggested that the contractility changes might be used for triggering a rate-adaptive pacemaker when cardiac pacing is indicated to prevent NMS (27). Later, the same authors performed a single-blind, randomized, crossover study comparing DDDR vs. DDI and concluded that, in patients with recurrent VVS, the symptomatic recurrences were less frequent during contractility-driven DDDR pacing than during DDI pacing (28).

The value of DDD stimulation with a CLS sensor in patients suffering from VVS was first described in 1998 (29). The reason for this benefit was assumed to be based on the CLS detecting the increase in contractility in the initial stage of VVS, which could activate the dual-chamber pacing that anticipates the large decrease in sympathetic tone and counteracts it, thus avoiding arterial hypotension, bradycardia, and possibly syncope. This hypothesis was supported by a study in which DDD-CLS significantly reduced the incidence of HUT-induced syncope when compared with DDD triggered by rate-drop-response. Pacing in DDD-CLS began 8 min earlier than in DDD, which may reflect sensing of reduction of the right ventricular volume occurring even before the rise in d*p*/d*t* (30). There remains a lack of sufficient data concerning the relative timing of blood pressure fall and epinephrine rise due to the epinephrine measurements being infrequent (24–26). However, it appears likely that the blood pressure fall due to reduced venous return precedes the contractility changes, and the CLS device is able to detect this (30). The early work of Italian researchers in a prospective registry showed encouraging results that heralded the value of this sensing system in VVS (31–33).

Since then, there have been six studies, some prospective, that have included patients with cardioinhibition during HUT, and all have suggested the usefulness of DDD-CLS stimulation to reduce the recurrence of VVS (summarized in Table 1). The first of these was the INVASY study, which was multicenter, prospective, randomized, and controlled but single-blind. It compared DDD-CLS stimulation with DDI mode at 30 bpm (essentially ineffective pacing), with the patients crossing over to the other stimulation mode after the second recurrence of syncope. DDD-CLS stimulation was more effective than DDI in preventing the recurrence of syncope during a mean follow-up of 19 months, and no recurrence was observed in the group of patients assigned to DDD-CLS (34) despite a number of protocol violations being there.

**Table 1:** Characteristics of studies using the DDD CLS mode in vasovagal syncope after HUT cardioinhibitory response.

**References**	**Methods**	**Blind**	**Patients**	**Follow-up (months)**	**Recurrences**
Occhetta et al. (34)	Multicenter, randomized, controlled, prospective	Single	50	19	DDI 78% DDD-CLS 0%
Kanjwal et al. (35)	Single-center, non-randomized, retrospective		44	9	DDD-RDR 83% DDD-CLS 59%
Bortnik et al. (36)	Single-center, prospective		35	61	DDD-CLS 17%
Palmisano et al. (37)	Single-center, retrospective		41	53	DDD-RDR 38% DDD-CLS 4%
Russo et al. (38)	Single-center, prospective cross-over	Single	50	36	DDD-CLS off 16% DDD-CLS on 2%
Palmisano et al. (30)	Multicenter, prospective randomized	Single	30		HUT-induced syncope DDD 76.7% DDD-CLS 30%

In a retrospective North American study with 35 patients that received 44 devices, 12 received a standard stimulation mode (RDR or simple-rate hysteresis), and 32 were stimulated with a DDD-CLS unit, where the recurrence was less (59 vs. 83%) and the reduction in syncope burden was greater (25 vs. 84%, *p* = 0.002) in those stimulated with a DDD-CLS device (35). Bortnik et al. (36) reported a prospective study including 35 patents with VVS, 83% of whom became asymptomatic when stimulated in the DDD-CLS mode.

A further retrospective, single-center study included 41 patients, 25 of them with DDD-CLS pacemakers and 16 of them with DDD-RDR, and only one patient (4%) in the DDD-CLS group compared with six patients (38%) in the DDD-RDR group had a recurrence of syncope (37).

Another Italian group conducted a prospective, randomized, single-blind, and cross-sectional study with 50 patients, all with DDD-CLS pacemakers randomized to pacemaker-ON vs. pacemaker-OFF for 18 months in each mode, with a total follow-up of 36 months. They showed a reduction in the number of syncopes (2 vs. 15; *p* = 0.007) and presyncopes (5 vs. 30; *p* = 0.004) in patients when they were stimulated with CLS vs. when they were not stimulated (38).

The most recent work has also been multicenter, prospective, randomized, and single-blind, including 30 patients with cardioinhibition during HUT who had been previously implanted with a DDD-CLS pacemaker for VVS. All were subjected to two new HUTs with a week between them: one in DDD-CLS mode and the other in DDD mode. The patients were randomly and blindly assigned to two groups, where in one group the first HUT was performed in DDD-CLS (*n* = 15) and in the other in DDD (*n* = 15). Compared with DDD, DDD-CLS significantly reduced the incidence of HUT-induced syncope (30.0 vs. 76.7%, *p* < 0.001). In patients with syncope, the DDD-CLS stimulation significantly delayed the onset of syncope during HUT (from 20.8 ± 3.9 to 24.8 ± 0.9 min; *p* = 0.032).

## Spain Study

To try to answer all of the previous questions, in 2006 the Syncope Working Group of the Spanish Society of Cardiology designed a randomized, double-blind, cross-over, prospective, and multicenter study that has attempted to verify the value of the DDD-CLS pacemaker against the DDI mode at 30 bpm in patients with recurrent VVS. Fifty-four patients ≥40 years old with cardioinhibition on HUT were included, 46 of whom completed the protocol. The patients were randomized to either DDD-CLS pacing for 12 months followed by sham DDI mode pacing at 30 ppm for 12 months (group A) or sham DDI mode for 12 months followed by DDD-CLS pacing for 12 months (group B). The patients in both arms crossed over after 12 months of follow-up or when a maximum of three syncopal episodes occurred within 1 month. During 22 months of follow-up, there was an overall ≥50% reduction in syncopes in 29 patients. In 72% of patients with DDD-CLS therapy vs. 28% with DDI in group A and in all group B patients, a reduction of ≥50% of syncopes was demonstrated once they crossed over from DDI therapy to DDD-CLS during the second year (*p* = 0.0003). Four (8.7%) patients suffered syncope while stimulated in DDD-CLS vs. 21 (45.65%) patients when they were in DDI (hazard ratio 6.72, odds ratio 0.11; *p* < 0.0001). A Kaplan–Meier analysis showed a significant prolongation of time until the first syncope with DDD-CLS vs. DDI (*p* < 0.0001 in both groups). The study concluded that DDD-CLS reduces the syncope burden and prolongs the time until the first syncope recurrence by seven-fold in patients >40 years with recurrent syncope and cardioinhibition during HUT compared with back-up DDI pacing (39).

In addition to this study, our group has recently published a pre-specified SPAIN subanalysis on the quality-of-life (QoL) data of the SPAIN study. QoL was assessed using the Short Form-36 (SF-36) health survey before randomization (baseline) and at 12 and 24 months of follow-up. Each SF-36 domain was scored from 0 to 100, with 100 representing the best perception of QoL. The change in QoL relative to the baseline was assessed and compared between the pacing algorithms (DDD-CLS vs. DDI). The mean SF-36 scores were significantly increased from baseline on DDD-CLS pacing across eight domains with the exception of “bodily pain.” QoL was significantly improved with DDD-CLS in “general health,” “vitality,” and “emotional role” (change in score of 9.6, 9.8, and 15.2, respectively; *p* < 0.05). Comparing the two pacing algorithms, the mean SF-36 scores were higher in the DDD-CLS group compared with the DDI group for the eight domains, and the differences in “physical role,” “bodily pain,” and “vitality” were statistically significant.

The analysis of the component summary scores indicated that DDD-CLS positively impacted both the mental and physical components, with significant differences in the physical component score, when compared with the DDI group. This pre-specified analysis of QoL in the SPAIN trial clearly demonstrates that the reduction in syncope burden and the extended time to the first syncope recurrence promoted by DDD-CLS translate into a significant and clinically relevant improvement in QoL. The DDD-CLS improved the perception of patients across both mental and physical components (40).

A recent meta-analysis has examined eight controlled trials (including 291 patients) that evaluated the CLS pacemaker therapy in patients with vasovagal syncope and cardioinhibitoion during HUT. They found that the use of CLS pacing was associated with a reduced risk of syncope (OR 0.08; 95% CI 0.03–0.18; *I*^2^ 32%) and presyncope (OR 0.34; 95% CI 0.18–0.63; *I*^2^ 0.00%). Using proportion meta-analysis, the summary estimate of the proportion of cases that developed syncope during CLS pacing was similar between the RCTs and the prospective studies (3.2 and 3.1%, respectively). This is much lower than the rate of recurrence in the control arm of RCTs at 33.7%. The sensitivity analyses yielded similar results. The authors concluded that, for patients with recurrent cardioinhibitory syncope confirmed by HUT, CLS pacing reduces the recurrent syncope and may improve the quality of life. Based on the findings of this analysis, “it should be considered” for patients who meet these criteria (41).

A new randomized trial called BIOSync is currently underway, which includes patients with VVS and cardioinhibition on HUT and who are randomized to DDD-CLS ON vs. OFF, and is hoped to confirm the findings of SPAIN (42), the results of which are expected in 2021.

## Issue-3 vs. Spain

There are similarities and differences between the ISSUE-3 and SPAIN trials. SPAIN required asystole/severe cardioinhibition on HUT, but in ISSUE-3, HUT was not required. However, 87% of ISSUE-3 patients underwent HUT, allowing the data to be available for subsequent analysis. ISSUE-3 required the finding of asystole on ILR (Table 2). A question must be asked concerning why there were differences in the pacemaker efficacy between these two studies. Firstly, ISSUE-3 included patients that had experienced more than or equal to three syncopal episodes in the previous 2 years, while in SPAIN the patients had more than or equal to five episodes and more than or equal to two episodes in the past year; so the SPAIN patients were much more symptomatic.

**Table 2:** ISSUE-3 and SPAIN trials compared.

	**ISSUE-3**	**SPAIN**
Diagnostic tool	Implantable loop recorder	Head-up tilt table
Number of patients included	77	54
Design	Double-blind, randomized, placebo-controlled, and parallel	Double-blind, randomized, placebo-controlled, and cross-over
Pacing mode	DDD-rate drop response	DDD-closed loop stimulation
Follow-up (months)	24	12
Recurrence rate in placebo arm (%)	57	45.7
Recurrence rate in pacing arm (%)	25	8.7
Relative risk reduction (%)	57	89
Absolute risk reduction (%)	Unknown	37
NNT	Unknown	2.7

Secondly, the pacing mode was RDR in ISSUE-3, while SPAIN used the DDD-CLS mode. The recurrence rate in the paced arm was 25% in ISSUE-3, while it was only 8.7% in SPAIN. This suggests that the pacing mode was the main reason for the difference, but a randomized, controlled trial of the two pacing modes would be needed to conclude this point.

Finally, two other differences may have played a part in the different results between the two studies: parallel groups (ISSUE-3) vs. crossover (SPAIN) design and 24 (ISSUE-3) vs. 12 (SPAIN) months of follow-up. Both trial design features are important in a condition such as the vasovagal syncope with its infrequent but cluster-prone behavior. Both are relevant when comparing ISSUE-3 and SPAIN and future trial designs.

## Negative Aspects

There are potentially deleterious effects of the permanent stimulation using a rate-responsive mode in a population of relatively young patients. It is well-known that the patients may occasionally experience side effects related to the so-called hyperchronotropism induced by rate-responsive modes. Further, the very long-term use of pacemakers, again in a relatively young population, must be expected to show complications, such as lead failure and infection at generator change, with predictable adverse effects. Finally, the resolution of even severe symptoms is known to occur without a specific treatment in the medium-term follow-up (43).

## Conclusions

It appears that the dual-chamber pacing with closed-loop system sensing has advantages over the rate-drop-response in the effectiveness of treatment of older (>40 years) patients with severe recurrent vasovagal syncope. The mechanism may be such that the closed-loop system introduces pacing earlier in a vasovagal episode. Evidence is available for the earlier stimulation by CLS in the vasodepression phase of vasovagal syncope, while RDR must wait for the later onset of bradycardia (cardioinhibition). The timing of onset of pacing may be the critical discriminator.

## Author Contributions

All authors listed have made a substantial, direct and intellectual contribution to the work, and approved it for publication.

### Conflict of Interest

GB-E is principal author of SPAIN study that was financed by an unrestricted grant to the Spanish Society of Cardiology of Biotronik. GB-E received honorary as Speaker and Consultant by Biotronik. The remaining authors declare that the research was conducted in the absence of any commercial or financial relationships that could be construed as a potential conflict of interest.
